# Complete Genome Sequence of *Microbacterium* sp. Strain SGAir0570, Isolated from Tropical Air Collected in Singapore

**DOI:** 10.1128/MRA.00613-19

**Published:** 2019-08-22

**Authors:** Namrata Kalsi, Daniela I. Drautz-Moses, Akira Uchida, Rikky W. Purbojati, James N. I. Houghton, Caroline Chénard, Anthony Wong, Sandra Kolundžija, Megan E. Clare, Kavita K. Kushwaha, Alexander Putra, Nicolas E. Gaultier, Balakrishnan N. V. Premkrishnan, Cassie E. Heinle, Vineeth Kodengil Vettath, Ana Carolina M. Junqueira, Stephan C. Schuster

**Affiliations:** a Singapore Centre for Environmental Life Sciences Engineering, Nanyang Technological University, Singapore; b Departamento de Genética, Instituto de Biologia, Universidade Federal do Rio de Janeiro, Rio de Janeiro, Brazil; DOE Joint Genome Institute

## Abstract

Microbacterium sp. strain SGAir0570 was isolated from air samples collected in Singapore. Its genome was assembled using single-molecule real-time sequencing and MiSeq short reads. It has one chromosome with a length of 3.38 Mb and one 59.2-kb plasmid. It contains 3,170 protein-coding genes, 48 tRNAs, and 6 rRNAs.

## ANNOUNCEMENT

Microbacterium is an aerobic, Gram-positive genus of bacteria that belongs to the phylum *Actinobacteria* (1). Microbacterium spp. are yellow-pigmented coryneform rods (2, 3). Some species of *Microbacterium* are pathogenic (4, 5). The genus *Microbacterium* was proposed by Orla-Jensen in 1919 (6). Since then, *Microbacterium* species have been reported in a diverse range of habitats, such as landfill surface soil (7), salt-marsh plants (8, 9), mushrooms (10), soil (11), air (12), and seawater (13). *Microbacterium* spp. have also been encountered in human clinical specimens (1, 2, 14).

The strain SGAir0570 was isolated from a tropical air sample collected at St. John’s Island in Singapore (1.2233N, 103.84472E) using an Andersen single-stage impactor (SKC, Inc., USA) and impacted onto brain heart infusion agar (Becton, Dickinson, USA). Colonies were then isolated by culturing on Trypticase soy agar at 30°C, followed by overnight cultivation in lysogeny broth at 30°C. The liquid culture was then used for DNA extraction with the Wizard genomic DNA purification kit (Promega, USA). The SMRTbell template prep kit 1.0 from Pacific Biosciences (PacBio, USA) was used for long-read library preparation, followed by single-molecule real-time (SMRT) sequencing on the PacBio RS II sequencing platform. Short reads were generated on a MiSeq 300-bp paired-end run platform (Illumina, USA) using whole-genome shotgun libraries constructed with the Illumina TruSeq Nano DNA library preparation kit.

For the following analysis pipeline, all software was run with default settings, unless otherwise stated. Quality control of PacBio reads was performed using PreAssembler filter v1 from the Hierarchical Genome Assembly Process (HGAP) v3 (15) protocol implemented in the PacBio SMRT Analysis 2.3.0 package. For MiSeq reads, Cutadapt v1.8.1 (16) was used. HGAP3 (15) was also used for *de novo* assembly with 70,531 PacBio subreads. The assembly was polished with Quiver (15) and corrected by aligning 903,132 paired-end Illumina reads using Pilon v1.16 (17) (–tracks –changes –vcf –fix all –mindepth 0.1 –mingap 10 –minmq 30 –minqual 20 –K 47). The PacBio raw reads showed an *N*_50_ value of 17,359 bp. Contig lengths and GC content were obtained using the Quality Assessment Tool for Genome Assemblies (QUAST) (18). The assembly produced 2 contigs, one being a chromosome with 3,380,208 bp (224-fold coverage) and one being plasmid pSGAir0570_2 with a length of 59,203 bp (43.9-fold coverage). The GC contents for the chromosome and the plasmid were 70.1% and 66.3%, respectively. Circlator was used to assess the circularity of the chromosome and plasmid. It identified high-similarity overlaps of the contig sequence ends for both the chromosome and the plasmid.

Genome annotation was performed using NCBI’s Prokaryotic Genome Annotation Pipeline (PGAP) v4.2 (19). A total of 3,268 genes were found, including 3,170 protein-coding genes (PCGs), 6 rRNA genes (5S, 16S, and 23S rRNAs), 48 tRNAs, 3 noncoding RNAs, and 41 pseudogenes.

The plasmid’s annotation showed a significant number of proteins related to toxins, such as an RHS repeat-associated core domain that secretes an exotoxin, and the HicA family toxin. In addition, the plasmid contains proteins that confer its host resistance to oxidative stress by camphor, arsenic, and copper, as well as other environmental stressors. In addition, the ImmA peptidase gene is present in the plasmid that is present in a conjugative transposon and assists in horizontal gene transfer (20).

Taxonomic identification was done using average nucleotide identity (ANI) analysis with Microbial Species Identifier (MiSI) (21) against a database of 6,387 bacterial RefSeq genomes with a text filter for type, synonym type, and proxytype and subsequently uses the getorf program with -find 3 option. This resulted in 79.8% similarity and 20% alignment fraction to Microbacterium hominis. Due to low ANI scores, Phyla-AMPHORA (22) and 16S rRNA identification were also performed using MarkerScanner.pl with added -DNA flag and MarkerAlignTrim.pl with options -WithReference and -OutputFormat phylip. Phylotyping.pl was run with default parameters. These resulted in 96.8% identity to the genus *Microbacterium* (minimum confidence, 1.0) and 100% identity to the genus *Microbacterium*, respectively, leading to the assignment of this organism to the *Microbacterium* genus.

Functional annotation with Rapid Annotations using Subsystems Technology (RAST) (23–25) showed that most genes were associated with carbohydrates (360 genes), as well as amino acids and derivatives (312 genes). Furthermore, 11 were linked to carotenoids, suggesting that SGAir0570 is potentially pigmented. This could provide protection from UV light in the air. The strain might also be motile, as 32 genes for flagellar motility were detected.

### Data availability.

The complete genome sequences of *Microbacterium* sp. SGAir0570 and its plasmid have been deposited in DDBJ/EMBL/GenBank under accession numbers CP027929 and CP027930, respectively, and in the SRA under accession numbers SRR8894405 and SRR8894406, respectively.
